# Efficacy of Biologics in the Treatment of Primary Sclerosing Cholangitis Associated With Inflammatory Bowel Disease: A Systematic Review and Meta-Analysis

**DOI:** 10.7759/cureus.56182

**Published:** 2024-03-14

**Authors:** David Huynh, Denis Rubtsov, Myat Myat Khaing

**Affiliations:** 1 Gastroenterology and Hepatology, The Prince Charles Hospital, Brisbane, AUS

**Keywords:** liver enzymes, liver, biological therapy, inflammatory bowel disease, primary sclerosing cholangitis

## Abstract

This is the first systematic review and meta-analysis that aims to address the scarcity of research on the use of biological therapy in primary sclerosing cholangitis-inflammatory bowel disease (PSC-IBD) and the historical inadequacy of therapeutic options. Its purpose is to investigate this matter comprehensively and furnish guidance for clinical practice. Utilizing Embase, PubMed, Medline, and clinicaltrials.gov studies investigating the roles of biologics and antibiotics in PSC-IBD were identified. The systematic literature review encompassed articles published from inception through September 2023. Two independent reviewers assessed the articles, and methodological quality was gauged using Review Manager 5.4.2. Nine studies were included in the systematic review and meta-analysis. However, only four met the criteria for inclusion in the meta-analysis due to variability and availability of data; the remaining studies underwent descriptive analysis. Notably, infliximab, adalimumab, vedolizumab, and tofacitinib showed ineffectiveness in reducing cholestatic markers. This review underscores the limited impact of biological and small-molecule therapies on disease progression in PSC-IBD patients, signifying the need for further exploration and development of treatment modalities in this domain.

## Introduction and background

Primary sclerosing cholangitis (PSC) is a rare and intricate liver disorder characterized by progressive inflammation and fibrosis of the biliary ducts. Its notable association with inflammatory bowel disease (IBD), particularly ulcerative colitis (UC), is well recognized [1,2]. This relationship is considered pivotal in understanding PSC. Approximately 70-90% of PSC patients also present with UC, whereas only 1-5% of individuals with IBD develop PSC [3,4]. Although PSC can co-occur with Crohn’s disease (CD), this association is less common, affecting around 10-20% of PSC cases. Consequently, PSC is categorized as a hepatobiliary complication or an extraintestinal manifestation of IBD [5,6].

Although the precise pathogenesis of PSC remains incompletely elucidated, one proposed theory posits that activated gut mucosal lymphocytes traverse to the liver, precipitating focal inflammation and fibrosis within the large and/or small bile ducts. Elevated serum levels of tumor necrosis factor-alpha (TNF-α), a key mediator of cellular damage in numerous inflammatory conditions, have been observed in patients with both IBD and PSC [7,8].

Therapeutically, options for PSC are limited, with the widely used ursodeoxycholic acid (UDCA) mainly offering symptomatic relief without significantly impacting disease progression [9,10]. Despite its immune-mediated nature, immunosuppressive agents, such as corticosteroids, methotrexate, and tacrolimus, have shown limited efficacy in altering the disease course [11-13]. Consequently, there is a pressing need for novel therapeutic strategies for PSC-IBD aimed at enhancing long-term survival, reducing the risk of liver transplantation, and mitigating malignancy development [11-13].

In contrast, the therapeutic landscape for IBD encompasses various established biological agents, including anti-TNF-α drugs (infliximab, adalimumab, golimumab, and certolizumab), adhesion molecule antagonists (natalizumab and vedolizumab), oral Janus kinase (JAK) inhibitors (tofacitinib and upadacitinib), and anti-interleukin (IL)-12/23 agents (ustekinumab). Studies evaluating these biologic agents in PSC-IBD patients have yielded diverse outcomes [14-22].

Given the inflammatory nature of the disease, we aim to assess the therapeutic potential of different biologic treatments used in IBD on PSC. We conducted a systematic review of clinical trials investigating their efficacy in adult patients. Our objectives were to evaluate the impact of these treatments on (1) cholestatic liver function tests compared to baseline values, (2) patients’ symptoms, and (3) the safety profile and incidence of adverse effects associated with biologic therapies.

## Review

Methodology

The Preferred Reporting Items for Systematic Reviews and Meta-Analyses (PRISMA) instructions were followed when conducting this systematic review and meta-analysis. The rules outlined in the Cochrane Handbook for Systematic Reviews of Interventions were also considered.

Literature Search

Embase, PubMed, Medline, and clinicaltrials.gov were the databases that we used to identify the studies examining the role of biologics and antibiotics in PSC-IBD patients. The systematic literature intended to find articles published from inception through September 2023. We used the following search strategy: [(‘IBD’ OR ‘inflammatory bowel disease’ OR ‘UC’ OR ‘ulcerative colitis’ OR CD OR ‘Crohn’s disease’)] AND [‘PSC’ OR ‘primary sclerosing cholangitis’ OR ‘cholangitis’]. We combined these with the following search strings for biologic agent-related terms: extra-intestinal manifestation, infliximab, vedolizumab, golimumab, certolizumab, natalizumab, tofacitinib, ustekinumab, and anti- TNF-α agents. To find studies that were missed by the online electronic searches, a manual search was also done among the reference lists of all known relevant publications, prior systematic reviews, and review studies, which is known as the “snowball” method. Initial screening was done considering the title and abstract; afterward, full-text screening was applied for including eligible studies. The search was performed by two researchers. Any discrepancies in the study selection process were resolved by the third researcher.

Study Selection Criteria

Inclusion criteria: (1) Studies that included adult patients aged 18 years or older with IBD-PSC. (2) Studies that studied the effectiveness of biologics in IBD-PSC patients. (3) Studies that assessed the long-term outcomes in IBD-PSC patients treated with biologics after any duration of follow-up.

Exclusion criteria: Conference abstracts, studies in languages other than English, reviews, case series, letters to the editor, studies not reporting on outcomes of interest, studies with insufficient data, and the study that included pediatric population were excluded from the review.

The included studies were independently reviewed by two reviewers using a standardized template. In cases of disagreement or ambiguity, a third reviewer was consulted to discuss and resolve the issues, ensuring a thorough and unbiased review process.

Data Extraction

Two investigators independently extracted the data from included studies in a formatted data extraction sheet that included the following characteristics of the studies: first author, study design, study year, sample size, study country, age at IBD diagnosis, duration of IBD and PSC, outcomes of interest including liver enzymes, follow-up duration, biologics studied, and concomitant treatments. We extracted data on liver enzymes and bilirubin before and after different biologics therapy for the meta-analysis.

Quality and Risk of Bias Assessment

Two authors performed the quality assessment of the included studies in accordance with the following items: (1) clarity of the study objectives; (2) study period stated clearly; (3) criteria for patient selection; (4) study conducted in multiple centers; (5) biologic treatment method and dosage mentioned; (6) baseline equivalence groups considered; (7) the definition of the primary outcome defined before the study; (8) adequate follow-up period; (9) adverse reactions stated; and (10) the limitations of each study were considered. We did not use quality assessment as an exclusion criterion. Individual study questions were answered with “yes” or “no,” and 1 point was awarded for “yes” and 0 points for “no.” A total score was calculated for each study. The quality of the included studies was judged to be fair for a total score of 8-10, average for 5-7, and low for 0-4.

Data Synthesis

We reported the standard mean differences of the variables such as alkaline phosphatase (ALP), aspartate aminotransferase (AST), alanine transaminase (ALT), and the serum total bilirubin before and after the different biologic therapy in IBD-PSC patients. Similarly, we calculated the standard mean differences of the values of the similar variables between the different biologic agents. Version 5.4.2 of ReviewManager for Windows was utilized. A 95% confidence interval (CI) was used in the interval estimation. When there was a lack of heterogeneity predicted by I^2^ ≤50% and p ≥0.05, the fixed-effect model was employed to combine the effect value. When there was heterogeneity suggested by I^2^ equal to or more than 50% and p <0.05, the random-effect model was applied. The publication bias was assessed by funnel plot of effect size against standard error for each study. The two-tailed chi-square test was used to test the hypothesis, and tau2 was estimated using DerSimonian and Laird method to verify the heterogeneity. An inverse-variance method was used to calculate the pooled standardized mean difference (SMD), expressed as a 95% CI. For interpretation, forest plots were created. Statistical significance was defined as a p-value of less than 0.05. SMD was used instead of mean difference because of the difference in measurement of the variables such as ALP. Moreover, the data in the median-interquartile range (IQR) was converted into mean and SD for the meta-analysis using the calculator suggested by a group of researchers [23-26].

Outcomes Assessment

The primary outcomes assessed were the effect of different biologics and antibiotics on liver function tests (ALP, AST, ALT, gamma-glutamyl transferase (gamma-GT), total bilirubin), degree of biliary tree dilatation and strictures, and elastography score. The secondary outcome was the long-term outcomes (incidence of cholangiocarcinoma, colorectal carcinoma, liver transplantation) among patients on biological treatment compared to patients on non-biological treatment.

Results

Search Results and Study Selection

**Figure 1:**
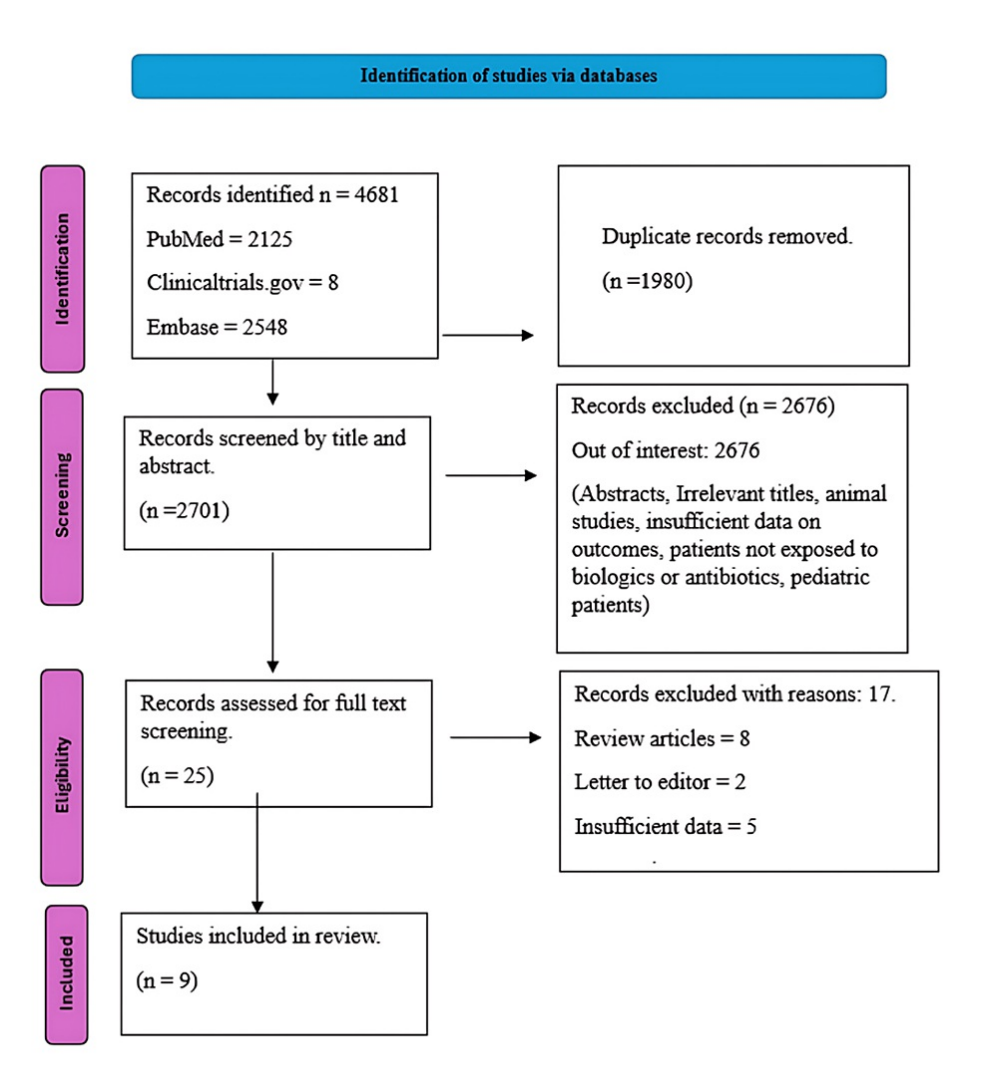
Preferred Reporting Items for Systematic Reviews and Meta-Analyses diagram illustrating the study retrieval process.

The initial literature search yielded 4,681 articles which was followed by the removal of duplicates. The remaining articles then were screened for the title and abstract. Further, the articles were screened as per our inclusion and exclusion criteria. To determine the eligibility for inclusion, the remaining 25 articles were read in full. Finally, the review included eight studies. Figure 1 elaborates the PRISMA flowchart illustrating the research search and selection procedure.

Study Characteristics

Among the nine included studies, one was a randomized study, and the majority were retrospective cohort studies. The efficacy of vedolizumab was evaluated in six studies. Similarly, adalimumab, infliximab, and golimumab were evaluated in four, five, and one studies, respectively. All studies were from European and North American countries. All studies were conducted between 2016 and 2022. The detailed demographics and the outcomes of interest of the included studies are depicted in Table 1.

**Table 1:** Characteristics and main outcomes of the included studies. *: mean ± SD. IBD: inflammatory bowel disease; PSC: primary sclerosing cholangitis; IQR: interquartile range; UC: ulcerative colitis; CD: Crohn’s disease; ALP: alkaline phosphatase; ALT: alanine transaminase; γ-GT: gamma-glutamyl transferase; CRP: C-reactive protein; IBD-U: IBD-unclassified; 5ASA:5-aminosalicylic acid; ULN: upper level normal; HR: hazard ratio

Study	Study country	Study design	IBD-PSC patients	Patients’ age at diagnosis (median IQR)	Gender (M:F)	Biologics studied	Concomitant treatments	Outcomes of interest	Follow-up (median, IQR) months	IBD duration (median, IQR) years	PSC duration (median, IQR)	Efficacy of treatments
Caron et al. 2019 [15]	France, Belgium	Retrospective, observational, multicenter study	All = 75, UC = 49, CD = 26	IBD: 20.9 (16.6–34.2). PSC: 26.0 (18.3–38.4)	51:24	Vedolizumab	Glucocorticoids 38 (51%). Immunosuppressants 23 (31%). Ursodeoxycholic acid 65 (87%)	Changes in the ALP, total bilirubin, ALT, AST, γ-GT, CRP, and serum albumin concentration	19.2 (14.4–30)	6.2 (3.7–13.5)	2.9 (0.4–6.6)	(Mean change (ULN), p-value). ALP: Week 30 +0.1; p = 0.66. Week 54 -0.01; p = 0.98. AST: Week 30 +0.1; p = 0.58. Week 54 -0.2; p = 0.33. ALT: Week 30 -0.01; p = 0.96. Week 54 -0.4; p = 0.22. Total bilirubin: Week 30 +0.5; p = 0.51. Week 54 +0.1; p = 0.84
Christensen et al. 2018 [16]	United States, Australia	retrospective cohort study	IBD = 34, UC = 18, CD = 16	CD = 19.5 (17–24). UC = 22 (18–39)	24:10	Vedolizumab	Tacrolimus: CD (13%) and UC (39%). Immunomodulator: CD (38%) and UC (39%). Glucocorticoids: CD (25%) and UC (44%). Antibiotics CD (6%) and UC (6%)	Changes in the ALP, total bilirubin, ALT, AST, γ-GT, CRP, and serum albumin concentrations	9 (7–16)	CD 10.5 (7.5–18.5). UC 10 (3–15)	CD 8 (3–10). UC 3 (1–8)	(IU/L, median, IQR) ALP baseline: 268 (105–551). Week 14: 265 (176–508) Week 30: 236 (183–634). AST Baseline: 54 (27–98). Week 14: 37 (23–75). Week 30: 46 (39–93). ALT Baseline: 63 (20–144). Week 14: 50 (31–107). Week 30: 58 (39–154). Bilirubin Baseline: 0.6 (0.4–0.9). Week 14: 0.7 (0.4–1). Week 30: 0.7 (0.4–1.3). Mayo PSC Risk Score (Mean (95% CI)) Baseline -0.40 (-0.85–0.05). Week 30 -0.38 (-0.83–0.08), p = 0.879
Franceschet et al. 2016 [17]	Italy	Prospective	All = 49, UC = 38, CD = 10, indeterminate colitis = 1	*PSC: 29.2 ± 14.8	29:20	Adalimumab, infliximab	Mesalazine, prednisone, azathioprine, mycophenolate mofetil	Liver function test, Mayo Score, Modified Truelove–Witts Severity Index, Harvey–Bradshaw Severity Index	*113.8 ± 90.3	NA	NA	Adalimumab decreased ALP at 6 and 12 months in two of the three patients treated. Infliximab reduced γGT at 6 and 12 months in one patient
Hedin et al. 2020 [18]	12 countries in Europe and North America	Retrospective study	All = 141, UC = 84, CD = 52, IBD-U = 5	IBD = 20 (15–30). PSC = 27 (20–38)	89:52	Infliximab, Adalimumab	UDCA, 5ASA, cortisone, immunosuppressants	Liver function test, PSC symptoms frequency	12 months	NA	NA	Infliximab ALP × ULN (IQR) (A) Baseline: 1.3 (0.8–2.4) 3 months: 1.2 (0.7–2.1) N = 66, p = 0.306. (B) Baseline: 1.4 (0.8–2.8) months: 1.4 (0.7–2.6) N = 62, p = 0.934. (C) Baseline: 1.4 (0.7–2.8) 12 months: 1.4 (0.7–2.6) N = 51, p = 0.106. Adalimumab ALP × ULN (IQR) (A) Baseline: 1.1 (0.7–2.4) 3 months: 0.9 (0.6–1.6) N = 23, p = 0.001. (B) Baseline: 1.1 (0.7–2.5) months: 0.7 (0.6–1.9) N = 18, p = 0.004. (C) Baseline: 1.1 (0.7–2.1) 12 months: 0.8 (0.6–1.5) N = 14, p = 0.011
Hommes et al. 2008 [19]	The Netherlands	A double-blind, placebo-controlled, randomized Study	6	?	4:2	Infliximab	Mesalazine, corticosteroids, azathioprine, ursodeoxycholic acid	Serum ALP, PSC symptoms	52 weeks	*14.7 ± 14.2	*7.5 ± 3.4	ALP (U/L), Mean infliximab group: Week 0: 349; week 18: 330; week 52: 389. Placebo group: Week 0: 481; week 18: 438; week 52: 391. PSC symptoms: Less in the infliximab group
Lynch et al. 2019 [20]	20 centers across Europe and North America	Retrospective analysis	All = 102, UC = 66, CD = 30, IBD-U = 6	*IBD: 26.0 ± 12.3 PSC: 31.4 ± 14.2	64:38	Vedolizumab	None	Liver biochemistry	46.75 (27–65.8)	NA	NA	Values are in median IQR ALP (IU/L × ULN) Baseline: 1.54 (0.86–2.67), n = 102. Last follow-up: 1.64 (1.04–3.47) ; p = 0.018, n = 102. AST (IU/L) Baseline: 38 (23–69), n = 102. Last follow-up: 49 (27–93); p = 0.0002, n = 68. ALT (IU/L) Baseline: 38 (22–76), n = 102. Last follow-up: 53 (29–100); p = 0.0002, n = 101. Bilirubin (mmol/L) Baseline: 10 (6.6–16.0), n = 102. Last follow-up: 12 (8–21); p = 0.0002, n = 97
Tse et al. 2018 [21]	United States	Retrospective cohort study	All = 88, UC = 55, CD = 30, Indeterminate = 3	IBD: Infliximab 22 (9–65). Adalimumab 26 (15–48). Vedolizumab 18 (12–67)	56:30	Infliximab, adalimimab, vedolizumab	Mercaptopurine, azathioprine	Liver biochemistries, biliary sclerosis, and hepatic stiffness scores before and after biological initiation	12–14 months			Vedolizumab (mean change, p-value) ALP M6 +50 U/L; p = 0.11. Month 12 +58 U/L; p = 0.24. AST M6 -2 U/L; p = 0.90. Month 12 -1 U/L; p = 0.98. ALT M6 -8 U/L; p = 0.78. Month 12 +0 U/L; p = 0.99. Total bilirubin M6 -0.5 μmol/L; p = 0.46. Month 12 +0.4 μmol/L; p = 0.70. Adalimumab (mean change, p-value) ALP Month 6 -70 U/L; p = 0.003. Month 12 -105 U/L; p = 0.052. AST M6 +1 U/L; p = 0.89. Month 12 +4 U/L; p = 0.65. ALT Month 6 -6 U/L; p = 0.58. Month 12 -11 U/L; p = 0.18. Total bilirubin Month 6 +0.1 μmol/L; p = 0.47. Month 12 -0.1 μmol/L; p = 0.30. Infliximab (mean change, p value) ALP Month 6 +37 U/L; p = 0.23. Month 12 +8 U/L; p = 0.89. AST Month 6 +9 U/L; p = 0.13. Month 12 +23 U/L; p = 0.22. ALT Month 6 +2 U/L; p = 0.90. Month 12 +11 U/L; p = 0.61. Total bilirubin Month 6 +0.2 μmol/L; p = 0.06. Month 12 +0.1 μmol/L; p = 0.31
Biron et al. 2022 [14]	France	Cohort study	IBD = 1,929, UC = 1,025, CD = 904		1,085:844	Infliximab, adalimumab, golimumab	-	Biliary tract cancer and liver transplantation	10 years	*6.9 ± 6.8	*2.2 ± 2.6	Patients exposed to anti-TNF agents did not have an elevated risk of biliary tract cancer compared to those not exposed to anti-TNF (HR, 0.59; 95% CI, 0.13-2.80). Patients exposed exposed to anti-TNF showed no elevated risk of liver transplantation (HR, 0.68; CI, 0.22-2.09)
Schrege et al. 2023 [22]	13 centers from Europe, North America, and the Middle East	Retrospective study	42 IBD-PSC patients	PSC: 28 (24.25) Median IQR	29:13	Tofacitinib	UDCA, biologics	Liver, bowel outcomes; Mayo Endoscopic Subscore and PSC outcomes, ALP, bilirubin	12 months	NR	NR	Longer treatment time with tofacitinib was associated with lower ALP ratio (baseline/last follow-up under treatment with tofacitinib) (r = -0.423; p = 0.011; n = 35) Liver stiffness remained stable in those with measurements before and under tofacitinib treatment (median, 5.0 kPA (IQR, 5.4 kPa) vs. 6.1 kPA (IQR, 5.6 kPa); p = 0.278; n = 13). Colitis activity significantly improved in the majority of patients (58%) assessed by the full Mayo Score (p = 0.003; n =27) as well as by mucosal appearance using the Mayo Endoscopic Subscore (p = 0.024; n = 33)

Patient Characteristics

The review included 2,466 IBD-PSC patients with 1,335 UC, 1,068 CD, 4 intermediate, and 59 unclassified cases. Among them, 1,432 (58.1%) were male patients. Concomitant treatments varied by the studies and included steroids, tacrolimus, UDCA, mesalazine, azathiopurine, mycophenolate mofetil, mercaptopurine, and 5-aminosalicylic acid. The follow-up duration ranged from 1 to 10 years. The median duration of IBD and PSC was above six and two years, respectively. The median age at IBD and PSC diagnosis was above 19 and 26 years, respectively. Table 2 shows the quality assessments of the included articles.

**Table 2:** Quality assessment of the included articles/studies.

Study	Clarity of the study objectives	Study period stated	Criteria for patient selection	Study conducted in multiple centers	Biologic treatment method and dosage mentioned	Baseline equivalence groups are clearly considered	Definition of the primary outcome	Adequate follow-up period	Adverse reactions stated	Limitations of each study were considered	Total score
Caron et al. 2019 [15]	1	1	1	1	1	1	1	1	1	1	10
Christensen et al. 2018 [16]	1	1	1	1	1	1	1	1	1	1	10
Franceschet et al. 2016 [17]	1	1	1	0	1	1	1	1	0	1	8
Hedin et al. 2020 [18]	1	1	1	1	1	1	1	1	1	1	10
Hommes et al. 2008 [19]	1	1	1	0	1	1	1	1	1	1	9
Lynch et al. 2019 [20]	1	1	1	1	1	1	1	1	0	1	9
Tse et al. 2018 [21]	1	1	1	0	1	1	1	1	0	1	8
Biron et al. 2022 [14]	1	1	1	0	1	1	1	1	0	1	8
Schregel et al. 2023 [22]	1	1	0	1	1	1	1	0	1	1	8

Outcomes of Interest

Due to constraints related to data availability and similarity among studies, our meta-analysis was limited to incorporating only four studies. The remaining studies that did not meet these criteria were included in a descriptive analysis. Not all studies had the same outcome measures for the different biologics.

Effect on Liver Enzymes

**Figure 2:**
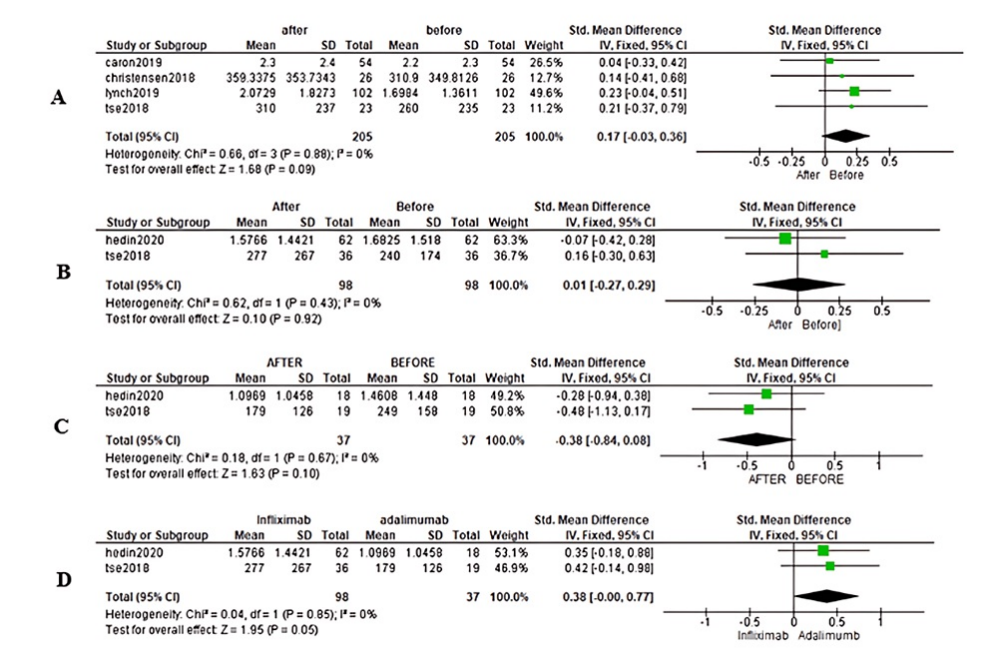
Forest plot of alkaline phosphatase (ALP) changes. (A) Forest plot showing ALP level before and after six months of therapy with vedolizumab. (B) Forest plot showing ALP level before and after six months of therapy with infliximab. (C) Forest plot showing ALP level before and after six months of therapy with adalimumab. (D) Forest plot showing ALP level after six months of therapy with adalimumab and infliximab [15,16,18,20,21].

ALP level: Figure 2 illustrates the pooled analysis of biological therapy effects on ALP levels. In the pooled analysis of four studies encompassing 205 patients, no significant difference in ALP levels was observed between baseline and after six months of vedolizumab therapy. The SMD was 0.17, with a CI of [-0.03, 0.36], I^2^ of 0%, and a p-value of 0.09. Similarly, in the pooled analysis of two studies involving 98 patients treated with infliximab, no significant difference in ALP levels was found between baseline and after six months of therapy, with an SMD of 0.01 (CI [-0.27, 0.29]), I^2^ of 0%, and a p-value of 0.92. Notably, although there was a reduction in ALP levels following six months of adalimumab therapy, this reduction did not reach statistical significance (SMD = -0.38, CI [-0.84, 0.08], I^2^ = 0, p = 0.10). When comparing ALP levels at six months between patients treated with infliximab and adalimumab, no significant difference was observed (SMD = 0.38, CI [-0.00, 0.77], I^2^ = 0, p = 0.05).

Although studies on tofacitinib could not be included in the meta-analysis due to differing data parameters, this medication appeared to demonstrate some reduction in baseline ALP levels. In the study conducted by Schregel et al. [22], the initial baseline ALP was recorded at 276 U/L. The results indicated an estimated marginal decrease of -68 U/L in the group that continued the medication compared to a +39 U/L increase in the group that discontinued treatment.

**Figure 3:**
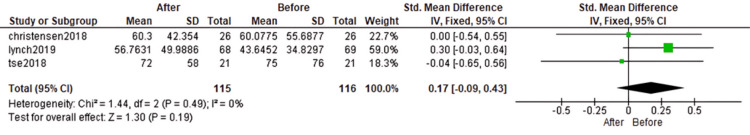
Forest plot showing aspartate transaminase level before and after six months of therapy with vedolizumab. [16,20,21].

AST level: Figure 3 demonstrates the AST levels before and after six months of vedolizumab. In the pooled analysis of three studies including 116 patients, there was no significant difference in the AST level between baseline and after six months of therapy with vedolizumab, with an SMD of 0.17, CI of [-0.09, 0.43], I^2^ of 0%, and p-value of 0.19.

**Figure 4:**
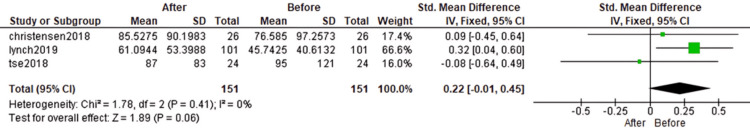
Forest plot showing alanine transaminase level before and after six months of therapy with vedolizumab. [16,20,21].

ALT level: In the pooled analysis of three studies including 151 patients, there was no significant difference in the ALT level between baseline and after six months of therapy with vedolizumab, with an SMD of 0.22, CI of [-0.01, 0.45], I^2^ of 0%, and p-value of 0.06 (Figure 4).

**Figure 5:**
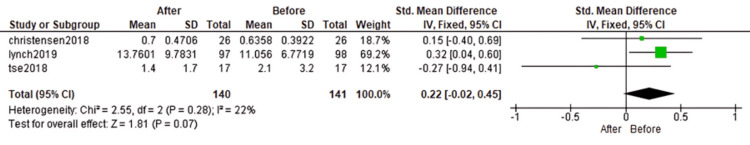
Forest plot showing total bilirubin level before and after six months of therapy with vedolizumab. [16,20,21]

Total bilirubin: Figure 5 shows the pooled analysis of three studies involving 141 patients. No significant difference in total bilirubin levels was observed between baseline and after six months of therapy with vedolizumab. The SMD was 0.22, with a CI of [-0.02, 0.45], I^2^ of 0%, and a p-value of 0.07.

In the study conducted by Hedin et al. [18], patients receiving infliximab exhibited an increase in bilirubin levels (0.33 times the upper limit of normal (ULN), IQR = 0.20-0.62) at baseline and follow-up (0.46 times ULN, IQR = 0.29-0.64, p = 0.003, n = 47), whereas no significant change was observed in patients receiving adalimumab. However, it is noteworthy that at baseline, recipients of infliximab had significantly lower bilirubin levels compared to those receiving adalimumab.

Effectiveness of Individual Biologics and Small-Molecule Medications in PSC

Vedolizumab: The evaluation of vedolizumab’s impact on liver enzymes was assessed across four studies, revealing no significant alteration in enzyme levels before and after therapy.

In the study by Caron et al. [15], it was found that 37%, 50%, and 46% of subjects had elevated serum ALP concentrations at Weeks 0, 30, and 54, respectively (>1.5 times the ULN; p = 0.24 and 0.52). Notably, 7% and 11% of patients experienced a reduction in baseline serum ALP levels of at least 50% by weeks 30 and 54, respectively. Among those with increased serum ALP concentration at week 0, no substantial change in any liver enzyme concentration was observed between week 0 and weeks 30 or 54, a trend also observed in patients with normal ALP concentration at week 0.

Christensen et al. [16] observed that among patients with elevated baseline ALP levels, 61% demonstrated improvement in ALP levels (from a median of 475 (IQR = 241-757) IU/L at baseline to 322.5 (IQR = 220-651) IU/L at week 14 (p = 0.025)). However, it is noteworthy that this improvement may be influenced by concurrent UDCA usage. Conversely, patients with normal baseline ALP levels experienced a significant increase in ALP levels at weeks 14 and 30. Further details on changes in liver enzymes are presented in Table 3, adapted from Christensen et al [16].

**Table 3:** Changes in liver tests. Adapted from Christensen et al. [16]. ALP: alkaline phosphatase; ALT: alanine aminotransferase; AST: aspartate aminotransferase; CI: confidence interval; IQR: interquartile range; PSC: primary sclerosing cholangitis

	Baseline	Week 14	P-value	Week 30	P-value
ALP (U/L), median (IQR)	268 (105–551)	265 (176–508)	0.35	236 (183–634)	0.99
Bilirubin (U/L), median (IQR)	0.6 (0.4–0.9)	0.7 (0.4–1.0)	0.62	0.7 (0.4–1.3)	0.96
AST (U/L), median (IQR)	54 (27–98)	37 (23–75)	0.22	46 (39–93)	0.69
ALT (U/L), median (IQR)	63 (20–144)	50 (31–107)	0.46	58 (39–154)	0.81
Mayo PSC Risk Score, mean (95% CI)	-0.40 (-0.85–0.05)	-0.59 (-0.99–0.18)	0.03	-0.38 (-0.83–0.08)	0.88

Lynch et al. [20] observed a 20% reduction in ALP levels in 20.6% (21) of individuals from baseline to the most recent follow-up. Conversely, ALP levels remained steady in 38.2% (39) of patients and increased by 20% in 41.2% (42) of patients at the last follow-up. Notably, the presence of cirrhosis emerged as the sole variable associated with a 20% decline in ALP from baseline to last follow-up in both univariate and multivariate analyses (odds ratio = 4.70; 95% CI = 1.61-13.76). Table 4, derived from Lynch et al., presents a detailed overview of changes in liver enzymes.

**Table 4:** Changes in liver tests. Adapted from Lynch et al. [20]. ALP: alkaline phosphatase; ALT: alanine aminotransferase; AST: aspartate aminotransferase; ULN: upper level normal; IQR: interquartile range

Liver function test median (IQR)	Baseline	Week 6	Week 14	Last follow-up
ALP (IU/L × ULN)	1.54 (0.86–2.67)	1.55 (0.82–2.95); p = 0.084	1.64 (1.00–3.61); p = 0.52	1.64 (1.04–3.47); p = 0.018
ALT (IU/L)	38 (22–76)	-	-	53 (29–100); p = 0.0002
AST (IU/L)	38 (23–69)	-	-	49 (27–93); p = 0.0002
Bilirubin (mmol/L)	10 (6.6–16.0)	-	-	12 (8–21); p = 0.0002

Adalimumab: Adalimumab demonstrated a tendency to reduce ALP levels, although this effect did not reach statistical significance. Notwithstanding, notable findings from the study conducted by Tse et al. [21] highlighted specific instances where adalimumab significantly influenced ALP levels. Over a treatment period of six to eight months, a noticeable decrease in ALP was observed (mean change of 70 U/L; SD = 88 U/L; p = 0.003). This trend persisted after 12-14 months of therapy, with a mean change in ALP of 105 U/L (SD = 148 U/L; p = 0.052). Interestingly, no significant alterations were reported in the levels of AST, ALT, total bilirubin, or direct bilirubin after 6-8 and 12-14 months of treatment (Table 5).

**Table 5:** Changes in liver tests. Adapted from Tse et al. [21]. ALP: alkaline phosphatase; ALT: alanine aminotransferase; AST: aspartate aminotransferase

Liver enzymes		Biologics			P-values for all biologics
At 6–8 months		Infliximab	Adalimumab	Vedolizumab	
ALP	N	36	19	23	
	Before	240 ± 174	249 ± 158	260 ± 235	0.92
	After	277 ± 267	179 ± 126	310 ± 237	0.18
	P-value	0.23	0.003	0.11	
AST	N	35	18	21	
	Before	39 ± 24	51 ± 32	75 ± 76	0.03
	After	48 ± 30	52 ± 33	72 ± 58	0.09
	P-value	0.13	0.89	0.90	
ALT	N	34	19	24	
	Before	51 ± 55	77 ± 62	95 ± 121	0.13
	After	56 ± 63	71 ± 56	87 ± 83	0.24
	P-value	0.90	0.58	0.78	
Total bilirubin	N	34	15	17	
	Before	0.7 ± 0.6	0.5 ± 0.4	2.1 ± 3.2	0.02
	After	0.9 ± 0.9	0.6 ± 0.3	1.4 ± 1.7	0.08
	P-value	0.06	0.47	0.46	
At 12–14 months					
ALP	N	26	10	19	
	Before	240 ± 174	249 ± 158	260 ± 235	0.92
	After	282 ± 321	171 ± 140	319 ± 228	0.36
	P-value	0.89	0.052	0.24	
AST	N	27	10	16	
	Before	39 ± 24	51 ± 32	75 ± 76	0.03
	After	65 ± 95	50 ± 31	80 ± 68	0.61
	P-value	0.22	0.65	0.98	
ALT	N	25	10	19	
	Before	51 ± 55	77 ± 62	95 ± 121	0.13
	After	67 ± 77	54 ± 39	103 ± 97	0.21
	P-value	0.61	0.18	0.99	
Total bilirubin	N	24	7	13	
	Before	0.7 ± 0.6	0.5 ± 0.4	2.1 ± 3.2	0.02
	After	0.8 ± 0.9	0.5 ± 0.3	1.0 ± 3.1	0.10
	P-value	0.31	0.30	0.70	

In a distinct investigation by Franceschet et al. [17], a reduction in ALP levels was observed, while gamma-GT levels fluctuated, and bilirubin remained stable at 6 and 12 months in two out of three patients treated with adalimumab. The study conducted by Hedin et al. [18] revealed that among 23 patients treated with adalimumab, there was a median reduction of 15% in ALP levels after three months. Among these patients, 10 individuals who had normal ALP levels at the initiation of treatment maintained their normal levels after three months of adalimumab therapy. Conversely, of the 13 patients with elevated ALP levels at baseline, 38% (5) of patients experienced a normalization of their ALP levels following treatment. For a more detailed breakdown of these changes in ALP levels, refer to Table 6.

**Table 6:** Changes in liver tests. Adapted from Hedin et al. [18] ALP: alkaline phosphatase

	Infliximab		Adalimumab	
	ALP × ULN, (IQR)	P-value	ALP × ULN, (IQR)	P-value
Baseline to 3 months	1.3 (0.8–2.4) 1.2 (0.7–2.1)	0.306 (n = 66)	(0.7–2.4) 0.9 (0.6–1.6)	0.001 (n = 23)
Baseline to 6 months	1.4 (0.8-2.8) 1.4 (0.7–2.6)	0.934 (n = 62)	(0.7–2.5) 0.7 (0.6–1.9)	0.004 (n = 18)
Baseline to 12 months	1.4 (0.7–2.8) 1.4 (0.7–2.6)	0.806 (n = 51)	(0.7–2.1) 0.8 (0.6–1.5)	0.011 (n = 14)

Moreover, patients treated with adalimumab exhibited a 33% reduction in ALP at 12 months compared to those treated with infliximab, after adjusting for elevated ALP at baseline and IBD response to the anti-TNF-α medication.

Infliximab: In the investigation conducted by Hedin et al. [18], findings demonstrated that among 67 patients treated with infliximab, there was a median decrease of 5% in ALP levels at three months. Among the 27 patients with normal ALP levels at baseline, six (22%) experienced elevated ALP after three months, while six (15%) of the 40 patients with elevated ALP at baseline saw their levels return to normal.

Tse et al. [21] reported no significant change in ALP levels at six to eight months (mean change = +37 U/L; SD = 183 U/L; p = 0.23). However, the use of infliximab was associated with a rise in direct bilirubin by +0.2 mg/dL (SD = 0.4 mg/dL) after six to eight months (p = 0.03). Furthermore, there were no notable alterations in AST and ALT levels after 6-8 and 12-14 months (refer to Table 5 for detailed data).

Additionally, the study by Hommes et al. [19] revealed that among six patients, one exhibited a 50% reduction in ALP levels. Mean ALP levels at weeks 0, 18, and 52 for infliximab-treated patients were recorded as 349, 330, and 389 U/L, respectively. Detailed data on changes in ALP levels are provided in Table 7, adapted from Hommes et al.

**Table 7:** Changes in alkaline phosphatase. Adapted from Hommes et al. [19]. *: At least a 50% decrease from screening.

	Placebo (n = 4)				Infliximab 5 mg/kg (n = 6)					
Patient number	101	104	106	109	102	103	105	107	108	110
Screening	529	256	478	914	862	378	437	232	238	940
Week 0	465	211	482	765	67*	412	330	207	225	854
Week 1	625		493	806	868	354	351	187	189	684
Week 2	472	221	410	821	634	355	274	171	152	587
Week 6	465	239	371	602	316*	362	347	190	207	503
Week 12	560	221	398	655	333*	625	239	233	150	530
Week 18	-	213	420	682	209*	334	273	260	-	572
Week 24	-	214	387	668	171*	-	256	-	-	580
Week 26	-		469	-	146*	331	290	357	-	481
Week 52	-	262	519	-	264*	500	548	242	-	-

Tofacitinib: Concerning tofacitinib, the meta-analysis was based on a single study, primarily due to limitations in data availability. In the study conducted by Schregel et al. [22], prolonged treatment with tofacitinib demonstrated a significant reduction in the ALP ratio from baseline to the latest follow-up (p = 0.011; n = 35). Upon adjusting for between-group variances, a statistically significant mean decrease in serum ALP from baseline during tofacitinib treatment was observed. Specifically, patients with a baseline ALP of 276 U/L exhibited a mean decrease of -68 U/L (95% CI = -132 to -4), contrasting with an increase of +39 U/L (95% CI = -27 to 104 U/L) observed in patients discontinuing treatment. Noteworthy, a correlation between the ALP response and IBD response to tofacitinib was also noted (p = 0.012).

PSC Symptoms

Only two studies have shed light on potential improvements in PSC-related symptoms. In the investigation by Hommes et al. [19], patients treated with infliximab exhibited milder symptoms of pruritus, fatigue, right upper quadrant pain, and weight loss compared to the placebo group. Similarly, Hedin et al. [18] reported that the prevalence of PSC symptoms showed no statistical difference between baseline and 12 months, except for abdominal pain, which became less common after 12 months compared to baseline. Notably, both infliximab and adalimumab demonstrated similar effects on the frequency of PSC symptoms following medication exposure.

Other PSC Outcomes

Not all studies comprehensively addressed various outcomes in PSC, such as risk scores or biliary dilatation. In the investigation by Tse et al. [21], conducted 6-12 months following the initiation of any biological therapy, no significant alterations were observed in elastography scores or radiographic imaging of biliary tree dilation/strictures. Following biological therapy, the majority of patients (88% treated with infliximab, 90% with adalimumab, and 79% with vedolizumab; p = 0.85) maintained stable biliary stenoses. Notably, only a small subset of six patients underwent magnetic resonance elastography before and after the initiation of biological therapy, with no significant changes detected in any of them.

In the studies by Biron et al. [14] and Christensen et al. [16], the computed Mayo PSC Risk Score demonstrated significant improvement from baseline to week 14, transitioning from a mean of -0.40 (95% CI = 0.85, 0.05) at baseline to -0.59 (95% CI = 0.99, -0.18) at week 14 (p = 0.03). However, by week 30, with a Mayo PSC Risk Score of 0.38 (95% CI = 0.83, 0.08), this difference was no longer statistically significant (p = 0.90).

Long-Term Outcomes and Adverse Events of Biologics and Small Molecules

Vedolizumab: Comparing the long-term outcomes and adverse events (AEs), in the study by Christensen et al. [16] 2018 that included 34 vedolizumab-receiving IBD-PSC patients, six patients stopped the drug due to no response and one for deranged liver function tests; all patients stopped the drug after a median of eight months. Furthermore, one underwent a colectomy, one had to opt for a liver transplant, and the other one had a dominant stricture. The other four minor events were headache, dental abscess, diarrhea, and upper respiratory infection.

In the study by Lynch et al. [20] including 105 patients, 20 (19%) patients had a three-fold increase in ALP, ALT, and AST. Moreover, in 21 (20%) patients, total bilirubin was doubled. During follow-up 561 days, there were eight liver transplantations, nine patients had at least one episode of cholangitis, and six experienced ascites, but none developed cholangiocarcinoma.

Meanwhile, in the study by Caron et al. [15] had 75 patients, 19 AEs, representing 25% of patients, occurred after a median follow-up of 1.6 years, including 11 (15%) severe AEs, which included infections in 10 cases and digestive neoplasia in nine cases (including seven colorectal and two cholangiocarcinoma).

Infliximab and adalimumab: Four studies reported AEs associated with the use of anti-TNFs. First, there were a few documented AEs by infliximab with the common symptoms being abdominal pain, pruritis, fatigue, dizziness, nausea, pyrosis, common cold, headache, and fever (Hommes et al. [19]). In the study by Hedin et al. [18], 49 (26%) of 186 patients had stopped anti-TNF-α (adalimumab and infliximab) due to AEs after a median of 539 days. The most common reason was non-response (n = 32, 17%) and AEs (n = 34, 18%) that included allergy, infection, skin disease, malignancy (n = 3), systemic lupus erythematosus, and recurrent cholangitis. In the study by Franceschet et al. [17], only one patient received infliximab and had rapid deterioration in liver function leading to a liver transplant.

Using the French national health insurance database, Biron et al. [14] conducted a population-based analysis of patients with PSC and IBD who were 18 years of age or older. The patients were monitored from January 1, 2009, to December 31, 2018. The risks of liver transplantation and biliary tract cancer linked to thiopurines and anti-TNF-α exposure (infliximab, adalimumab, and golimumab) were evaluated. There were 83 liver transplants and 37 biliary tract malignancies among the 1,929 patients with PSC and IBD who were included in the study. Patients exposed to anti-TNF-α agents did not have an elevated risk of biliary tract cancer compared to those not exposed to anti-TNF-α medicines (HR = 0.59; 95% CI = 0.13-2.80). Likewise, patients exposed to anti-TNF-α medications showed no elevated risk of liver transplantation (HR = 0.68; CI = 0.22- 2.09).

Tofacitinib: In a multicenter retrospective study, Schregel et al. [22] described the safety and efficacy of tofacitinib in patients with IBD-PSC. The median time of treatment was 20.4 months. It was reported that tofacitinib was well tolerated among the included cohort, with only two cases that had to be discontinued due to worsening colitis. Other AEs reported were minor, were not described, and did not lead to discontinuation; however, none had severe AEs such as thrombosis, reactivation of viral infections, or lymphoproliferative disorder.

Discussion

PSC represents a rare and enigmatic condition, often leading to elevated mortality rates or necessitating liver transplantation within 8-12 years post-diagnosis [27]. The rarity of this disease impedes large-scale randomized controlled trials (RCTs) investigating the efficacy of biological treatments. Emerging research has identified several factors, including genetic predisposition, immune-mediated pathways, bile acid imbalance, and gut dysbiosis, as potential contributors to the pathogenesis of PSC-IBD [28,29]. Given the limited therapeutic options, there is a burgeoning interest in exploring novel treatment avenues for this patient subset. The close epidemiological ties and shared immunopathogenesis pathways between PSC and IBD have fueled investigations into the effectiveness of biologics in treating PSC [21].

Our systematic review and meta-analysis represent the first comprehensive examination of the effects of biologics in adult IBD-PSC patients. We collated data from nine multicenter studies, encompassing a total of 2,466 IBD-PSC patients, including 1,335 with UC, 1,068 with CD, 4 intermediate cases, and 59 unclassified cases. Notably, the inclusion of more non-randomized studies than RCTs in our analysis limited the meta-analysis to just four of these articles.

Our hypothesis centered on the potential of blocking lymphocyte trafficking and shared pro-inflammatory molecules, such as TNF-α, to alter the trajectory of PSC in individuals with concurrent IBD. Among the various biologics examined, vedolizumab emerged with the most available data. However, our findings indicated that vedolizumab did not demonstrate efficacy in treating PSC. Specifically, neither the concentration of ALP nor the levels of other serum liver enzymes changed significantly in IBD-PSC patients following vedolizumab therapy. Moreover, subgroup analysis based on baseline ALP levels failed to reveal any difference in vedolizumab efficacy in PSC patients. Interestingly, the presence of cirrhosis, as highlighted in the study by Lynch et al. [20], was associated with a decline in ALP levels. However, the study by Christensen et al. [16] suggested that the decline in ALP after vedolizumab therapy might be confounded by concomitant UDCA use.

Our data indicates that biologics do not significantly alleviate cholestasis in IBD-PSC patients. Conversely, there was a modest increase in markers of cholestasis, including ALP, AST, ALT, and bilirubin levels, associated with the use of other biologics, except for adalimumab, which showed a decrease in ALP levels. Nonetheless, none of the studied biologics demonstrated a decrease in AST, ALT, or bilirubin levels. It is worth noting that the lack of improvement in liver function tests over the relatively short treatment duration may not necessarily indicate biologic ineffectiveness. Instead, it may suggest a halt in further PSC deterioration, albeit limited by the absence of proven surrogate markers for disease modification as well as limited imaging data in the studies. Additionally, the duration of biologic administration may have been insufficient to observe longer-term effects.

The emergence of small molecules, such as first and second-generation JAK inhibitors, presents a unique opportunity for treating IBD-PSC. Although data demonstrating the efficacy of these inhibitors in IBD patients are promising, their application in IBD-PSC remains limited [30,31]. A descriptive analysis of one study suggested that tofacitinib might be beneficial in reducing markers of cholestasis, although other indicators of disease progression, such as comments on biliary dilation and imaging, were not included. Nevertheless, data on JAK inhibitors are evolving, and they may offer disease-modifying treatment for IBD-PSC.

Despite the immunological underpinnings of PSC, the available biologics have shown limited ability to alter disease course or surrogate markers of disease progression. Antibiotics, however, have demonstrated promising results in improving these markers, suggesting a potential link between gut dysbiosis and PSC pathogenesis. Notably, a study by Shah et al. [22] revealed a significant reduction in ALP levels with vancomycin and metronidazole compared to the biologics assessed in our study, particularly at three months but with the same limitations described previously [32].

Limitations

Our systematic review and meta-analysis had certain limitations. First, the included studies were of non-randomized and retrospective design with a smaller sample size. We could include only nine studies in the systematic review and only four in the meta-analysis. Analysis was not possible for several studies because of different outcome measures, missing data, and few patients included. In addition, the results may also have been affected due to varied concomitant use of other treatments.

## Conclusions

Overall, our review underscores that biological and small-molecule therapies do not significantly modify disease courses in IBD-PSC patients. While biologics may potentially arrest disease activity, as indicated by delayed ALP elevation, questions remain regarding their disease-modifying effects in the absence of imaging data during therapy. With most biologics, except for adalimumab, demonstrating an increase in ALP levels, disease monitoring may pose challenges. Larger randomized clinical trials, with ample sample sizes and adequate follow-up durations, are warranted to further explore the effectiveness of biologics in IBD-PSC patients. The idea of contaminant treatment in PSC-IBD overlap still requires extensive research. At this stage, traditional treatment options remain the first-line therapy.
